# Association between marfan syndrome and oral health status: A systematic review and meta-analysis

**DOI:** 10.4317/medoral.23037

**Published:** 2019-06-25

**Authors:** Cosimo Galletti, Octavi Camps-Font, Gisela Teixidó-Turà, Inmaculada Llobet-Poal, Cosme Gay-Escoda

**Affiliations:** 1 DDS, MS. Master of Integrated Adult Dentistry. Associate professor of Integrated Adult Dentistry, Faculty of Medicine and Health Sciences, University of Barcelona, Barcelona (Spain); 2 DDS, MS. Master of Oral Surgery and Implantology. Associate Professor of Oral Surgery and Professor of the Master’s Degree Program in Oral Surgery and Implantology, Faculty of Medicine and Health Sciences, University of Barcelona, Barcelona (Spain); 3 MD, PhD. Department of Cardiology, Hospital Universitari Vall d’Hebron, University Autònoma de Barcelona (Spain); 4 NS. Department of Cardiology, Hospital Universitari Vall d’Hebron, University Autònoma de Barcelona (Spain); 5 MD, DDS, MS, PhD, EBOS, OMFS. Chairman and Professor of Oral and Maxillofacial Surgery, Faculty of Medicine and Health Sciences, University of Barcelona, Barcelona (Spain). Director of Master’s Degree Program in Oral Surgery and Implantology (EHFRE International University/FUCSO). Coordinator/Researcher of the Bellvitge Biomedical Research Institute. Head of Oral Surgery, Implantology and Maxillofacial Surgery Department of the Teknon Medical Center, Barcelona (Spain)

## Abstract

Background: The purpose was to identify and assess the existing scientific evidence from epidemiologic, non-experimental, observational studies of associations between Marfan’s syndrome and oral diseases. Material and Methods: Electronic literature searches in MEDLINE (OVID), The Cochrane Library, Scopus and the Web of Science were conducted to identify all relevant articles. Eligibility was based on inclusion criteria, and quality assessments were conducted. The outcome variables were probing depth, gingival margin, clinical attachment level, bleeding on probing, gingival status, periodontal status, tooth mobility, furcation involvement and decayed, missing and filled teeth index. After extracting data, meta-analyses were carried out. Results: Out of 527 potentially eligible papers, 3 cross-sectional studies were included. No statistically significant differences were found in the number of sites with bleeding on probing (OR: 1.26; 95% CI: 0.47 to 3.42; P = 0.65; I2: 0%), probing depth (MD: -0.14 mm; 95% CI: -0.24 to 0.53; P = 0.46; I2: 93%), periodontal status (WMD: 0.68 points; 95% CI: -0.48 to 1.83; P = 0.25; I2: 98%) nor number of decayed, missing and filled teeth index score (MD: 1.08 points.; 95% CI: -1.27 to 3.42; P = 0.37; I2: 0%). Conclusions: Patients diagnosed with Marfan’s syndrome do not seem to have worsened oral health status. Due to the high number of patients with Marfan’s syndrome that have prosthetic heart valves, an adequate dental monitoring as well as a strict maintenance therapy program should be implemented.

** Key words:**Marfan syndrome, oral health, periodontal diseases, caries.

## Introduction

Marfan’s syndrome (MFS) is a multisystem connective tissue disorder, first described more than 100 years ago by a Parisian professor of paediatrics, Antoine-Bernard Marfan (1). Its incidence is about 1 case per 5,000 individuals, although this figure may be underestimated (2).

This autosomal heritable disease is mainly attributable to a defect in the microfibrillar protein fibrillin-1 (FBN1) gene on chromosome 15 (15q21.1). This gene encodes FBN1, a matrix glycoprotein that is the main constituent of the microfibrils of the extracellular matrix (3,4). FBN1 monomers bond to form complex extracellular macroaggregates, called microfibrils, which form part of elastic fibers, and confer important biomechanical properties in connecting, anchoring, and maintaining tissues and organs (2). In addition, it has been proven that FBN1 stimulates the release and activation of TFG (a potent inflammation stimulator), fibrosis and it also activates certain matrix metalloproteinases (MMPs), especially MMP-2 ad MMP-9 (5).

MFS manifestations typically involve the cardiovascular, skeletal, and ocular systems. Cardiac disease is a predominant feature of MFS and includes proximal ascending aortic dilation, dilation of the proximal main pulmonary artery, thickening and prolapse of either or both atrioventricular valves, mitral annular calcification and, in some rare cases, dilated cardiomyopathy in the absence of severe valvular dysfunction (6). These complications are recognized as the major cause of morbidity and mortality in patients with MFS. In fact, aortic dissection or rupture account for most of the premature mortality among patients with MFS, a risk that increases rapidly during adolescence and results in death in up to 50% of undiagnosed and untreated patients with MFS by the age of 40. Disproportionate overgrowth of the long bones is often the most striking and immediately evident manifestation. Additional skeletal features in MFS include arachnodactyly (overgrowth of the fingers), joint hypermobility, anterior chest deformity and thoracolumbar scoliosis. With reference to the ocular manifestations, ectopia lentis (dislocation of the ocular lens) is the most common condition, affecting around 60% of patients with this disorder (2).

The diagnosis of MFS is challenging since many of its manifestations are present in other syndromes as well as in the general population. Although genetic tests are available, the diagnostic criteria of the current Ghent nosology still require clinical manifestations for final diagnosis (7).

In addition to the aforementioned multisystemic features, MFS also exhibits characteristic oral manifestations including retrognathia, dolichocephaly, high palatal vault, crowded teeth, temporomandibular joint disorders and partial anodontia (8,9). Moreover, in the presence of biofilm on the tooth surface, metabolic and compositional alterations of the periodontal ligament and/or the extracellular matrix may all have a substantial and negative impact on periodontal tissues, leading to increased susceptibility and trigger an inflammatory response that ultimately leads to tissue breakdown (10).

Nonetheless, little is still known about the oral health status of patients with MFS. Because these patients are cared for by dental professionals, it is essential to report on oral health features, particularly due to the aforementioned potential cardiovascular complications. Hence, the prevention of bacteremia caused by advanced tooth decay, pulpal infection and/or periodontal diseases should be prioritized in dental treatment planning.

Therefore, the aim of the present systematic review was to identify and assess the existing scientific evidence from epidemiologic, non-experimental and observational studies of associations between MFS and oral diseases.

## Material and Methods

-Protocol and registration

This paper adheres to the Preferred Reporting Items for Systematic Reviews and Meta-Analyses (PRISMA) declaration (11) and is registered in PROSPERO under number CRD42018115713.

-Eligibility criteria

The predefined study population (P), exposition (E), comparison (C), outcome parameters (O) and study type (S) (PECOS factors) for eligibility of the studies are summarized in  Table 1.

**Table 1:**
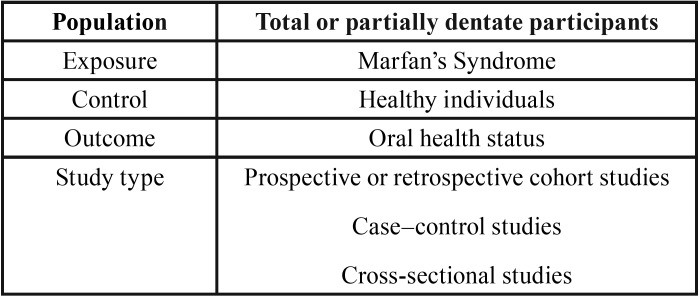
Issues of interest by study population (P), exposure (E), comparison (C), outcome (O) and study type (S) (PECOS factors).

Study populations needed to consist of total or partially dentate humans whose oral health status had been clinically assessed (population) in order for them to be included in the study. Cases had to be diagnosed according to Ghent Nosology for MFS criteria (7) (exposition) and a comparison group consisting of healthy individuals was included in the study (comparison). The results needed to include at least one of the following parameters related to oral health status reported at the patient level (outcomes):

• Probing depth (PD): The distance from the gingival margin to the tip of the periodontal probe assessed at six sites per tooth.

• Gingival margin (GM): The distance from the gingival margin to the cementoenamel junction (CEJ) assessed at six sites per tooth.

• Clinical attachment level (CAL): The probing depth plus the distance from the gingival margin to the CEJ assessed at six sites per tooth.

• Bleeding on probing (BOP): Presence or absence of BOP assessed at six sites per tooth.

• Löe-Silness Gingival Index (GI) (12).

• Periodontal status: Assessed by the Community Periodontal Index Treatment Needed (CPITN) (13), the Community Periodontal Index (CPI) (14) or the Periodontal Screening and Recording (PSR) index (15).

• Presence or absence of tooth mobility.

• Presence or absence of furcation involvement.

• Decayed, Missing and Filled Teeth Index (DMF-T) (16).

The inclusion criteria were original, prospective or retrospective non-interventional cohort, case–control, or cross-sectional studies exploring the status or evolution of periodontal health in humans known to have MFS.

The review excluded studies with less than ten patients in the control and/or exposure group.

-Search strategy 

An electronic search of the MEDLINE (OVID), The Cochrane Library (Wiley), Scopus (Elsevier) and the Web of Science (Thomson Reuters) databases up to September 1, 2018 was conducted in order to identify all relevant human studies without year or language restrictions.

For the PubMed library, the following research terms were applied: (“marfan’s syndrome”[MeSH Terms] OR “marfan syndrome”[MeSH Terms] OR “syndrome, marfan”[MeSH Terms] OR “syndrome, marfan’s”[MeSH Terms]) AND (((“stomatognathic diseases”[MeSH Terms] OR “oral manifestations”[Title/Abstract] OR “oral diseases”[Title/Abstract] OR “oral health”[MeSH Terms] OR “tooth diseases”[MeSH Terms]) OR (“dental caries”[MeSH Terms])) OR (“periodontal diseases”[MeSH Terms] OR “periodontitis”[MeSH Terms] OR “periodontal conditions”[Title/Abstract] OR “gingivitis”[MeSH Terms])). For searching the remaining electronic databases, the key terms used were: ‘marfan syndrome’ AND ‘oral health’ OR ‘tooth diseases’ OR ‘caries’ OR ‘periodontitis’ OR ‘gingivitis’ OR ‘periodontal diseases’.

Additionally, grey literature was searched on OpenGrey** as well as the US National Institutes of Health†† in order to identify additional potential candidates to be included. The research was completed by a manual screening of the references cited in the selected articles and reviews.

-Selection of studies

Two examiners (C.G. and O.C.F.) independently selected the studies in accordance with the inclusion criteria. Consensus resolved any disagreements.

**Figure 1:**
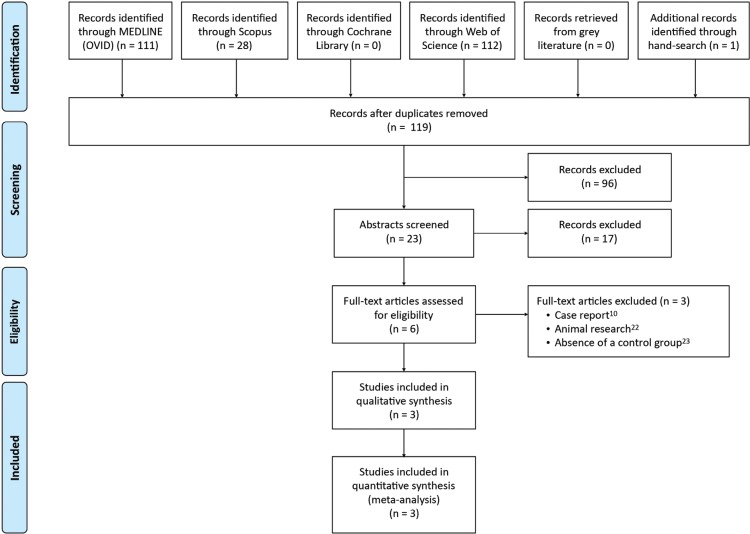
Flow-chart illustrating the study selection process.

Initially, duplicates or irrelevant publications (based on the title) were excluded, and the abstracts were examined. Finally, the full texts of all the remaining papers were assessed. The studies removed at this stage and the reasons for their exclusion were recorded (Fig. 1).

Authors were contacted when necessary for clarification of missing information. When multiple reports on the same patients were identified, only the most recent one was included.

-Data extraction and method of analysis

Two reviewers (C.G. and O.C.F.) independently extracted the data using data-extraction tables. Whenever possible, the following data were retrieved from the selected papers: author(s), year of publication, country of origin, study design and details of the participants and outcomes.

-Quality and risk of bias assessment

As part of the data extraction process, two reviewers (C.G. and O.C.F.) independently assessed the risk of bias of the RCTs included, using a modification of the Newcastle-Ottawa Scale (NOS) ‡‡.

** OpenGrey. Available at: www.opengrey.eu. Accessed September 1, 2018.

†† National Institutes of Health. ClinicalTrials.gov. Available at: www.clinicaltrials.gov. Accessed September 1, 2018.

‡‡ Wells G, Shea B, O’Connell D, *et al.* The Newcastle-Ottawa Scale (NOS) for assessing the quality of nonrandomised studies in meta-analyses. Available at: www.ohri.ca/programs/clinical_epidemiology/oxford.asp. Accessed 5 November, 2018.

The following items were evaluated: 1) selection of study groups, 2) comparability of the study groups, and 3) outcome. Each study received a maximum of 13 points for cohort studies, 10 points for case–control studies, and 7 for cross-sectional studies.

Authors were contacted for clarification of missing or unclear information when necessary.

-Statistical analysis

For dichotomous outcomes, odds ratios (OR) with 95% confidence intervals (95% CI) were used to estimate the effect of an exposition. Parametric and nonparametric tests (Pearson χ2, Fisher and Mann-Whitney tests) were used to compare the groups. For continuous outcomes, mean differences (MD) and standard deviations (SD) were used to summarize data for each group. The statistical unit was the patient.

A meta-analysis was only performed when studies reported the same outcome measures. Odds ratios and MD were combined for dichotomous and continuous data, respectively, using random-effects models. The random model was selected because it is more general than fixed effects models and we assumed heterogeneity between studies a priori. Statistical significance was defined as *P* <.05 for all analyses.

Statistical heterogeneity was estimated by means of χ2 (Q value) and I2 analyses. A χ2 *P*-value of <.10 and an I2 value of >50% were interpreted as significant heterogeneity (17).

Had there been a sufficient number of meta-analyzed studies (more than 10), publication bias, clinical heterogeneity assessment and sensitivity analyses would have been performed according to Patsopoulos *et al.* (18).

The statistical analysis was carried out using Review Manager software (Review Manager version 5.3; The Cochrane Collaboration, Copenhagen, Denmark).

## Results

-Study selection and description

The initial electronic database and gray literature research yielded 248 references. After duplicate removal and assessment of both titles and abstracts, a total of 6 articles were eligible for full-text analysis (Fig. 1). The reviewers’ agreement was 100%, with a κ index of 1 (perfect agreement).

After applying the study criteria, 3 publications were excluded because of case report (10), animal research (19), and absence of a control group (20), respectively.

Finally, 3 cross-sectional studies fulfilled the inclusion criteria and were selected for qualitative and quantitative synthesis (8,9,21) ( Table 2).

**Table 2:**
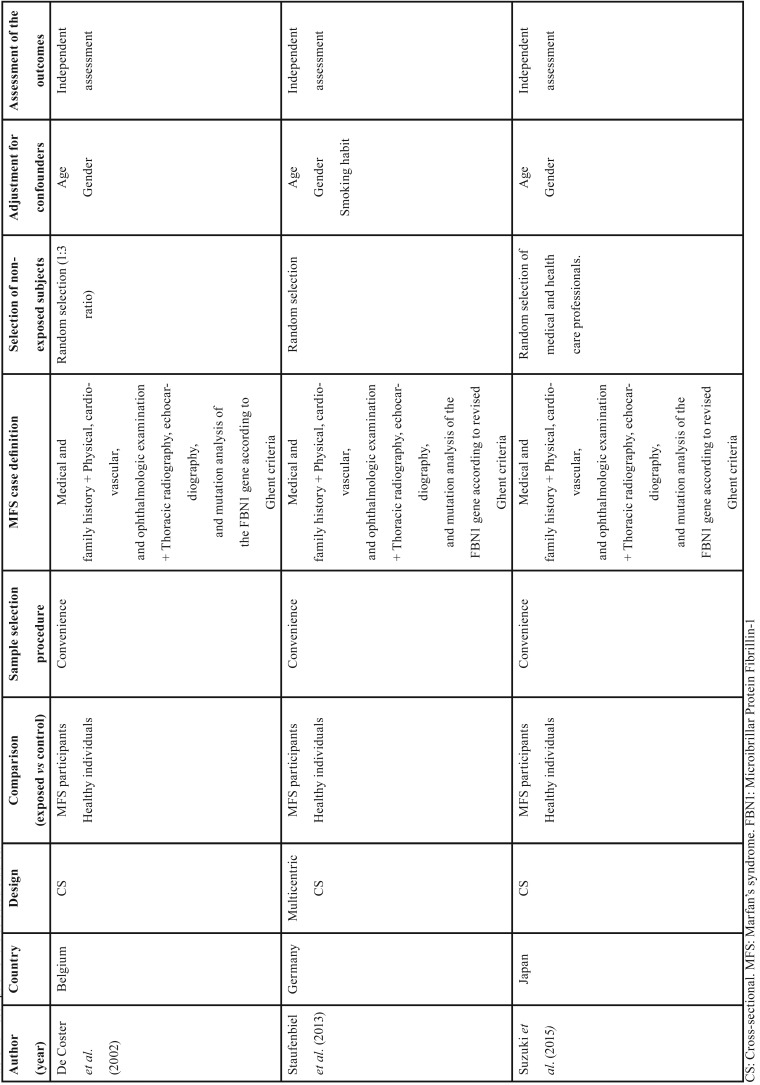
Description of the selected studies.

Figure 1 shows a flowchart of the screening process.

-Risk-of-bias assessment

All 3 studies were assessed by the modified and adapted NOS. The mean NOS score was 4 (Range: 3 to 5), being the domain “Selection” the highest ranked and the “Outcome” the lowest ( Table 3).

**Table 3:**
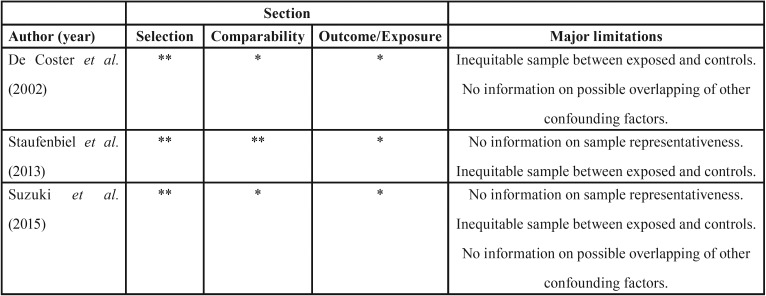
Quality assessment of 3 studies included in the qualitative evaluation according to the modified Newcastle-Ottawa Scale (NOS).

-Extraction data

°Qualitative synthesis

The three studies selected comprised 228 patients, 114 of whom were diagnosed of MFS and the rest served as controls ( Table 4,  Table 4 continue) (8,9,21).

**Table 4:**
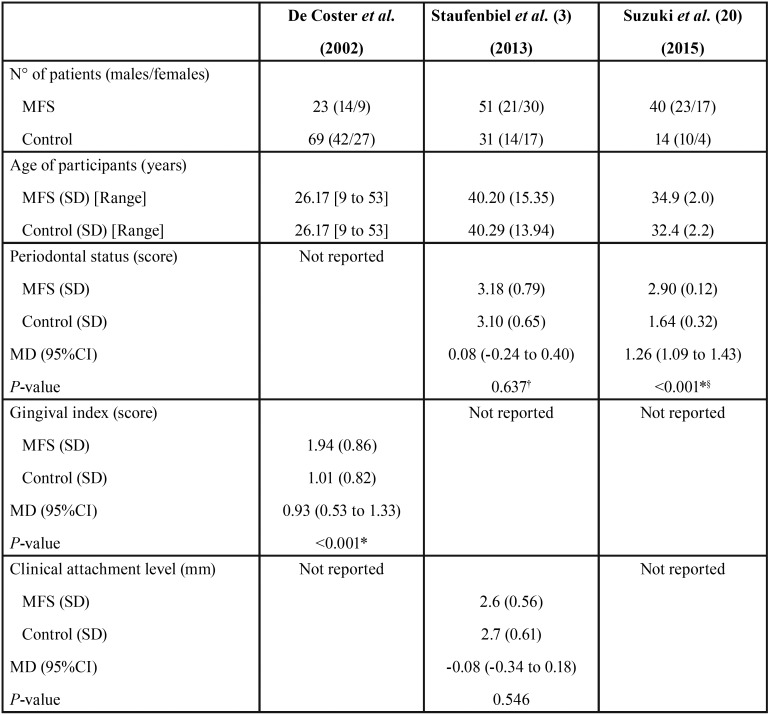
Comparison of the selected studies.

**Table 4 continue:**
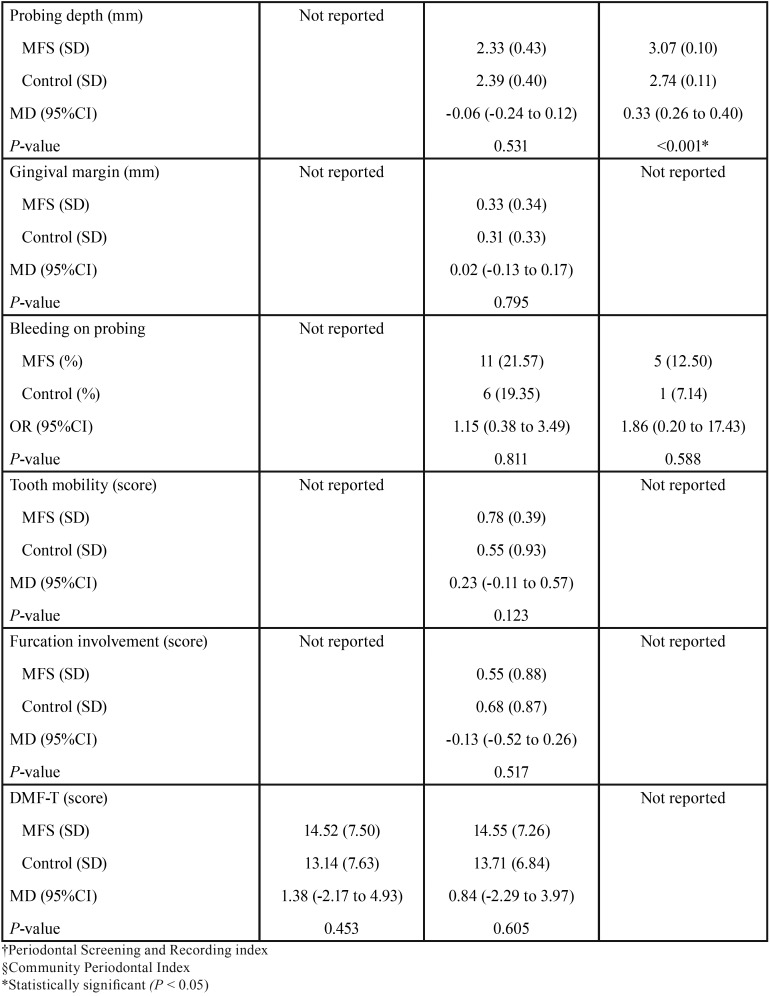
Comparison of the selected studies.

None of the studies revealed significant differences between the groups in terms of CAL, GM, BOP, GI, tooth mobility, furcation involvement nor DMF-T index score (*P* > 0.05) ( Table 4,  Table 4 continue).

Suzuki *et al.* (21) found higher PD (MD: 0.33 mm; 95% CI: 0.26 to 0.40; *P* < 0.001) as well as CPI scores (MD: 1.26 points; 95% CI: 1.09 to 1.43; *P* < 0.001) in patients with MFS ( Table 4,  Table 4 continue). Additionally, one paper reported worsened gingival conditions among MFS participants (MD: 0.93 points; 95% CI: 0.53 to 1.33; *P* < 0.001) ( Table 4,  Table 4 continue) (9).

°Quantitative synthesis

**Figure 2:**
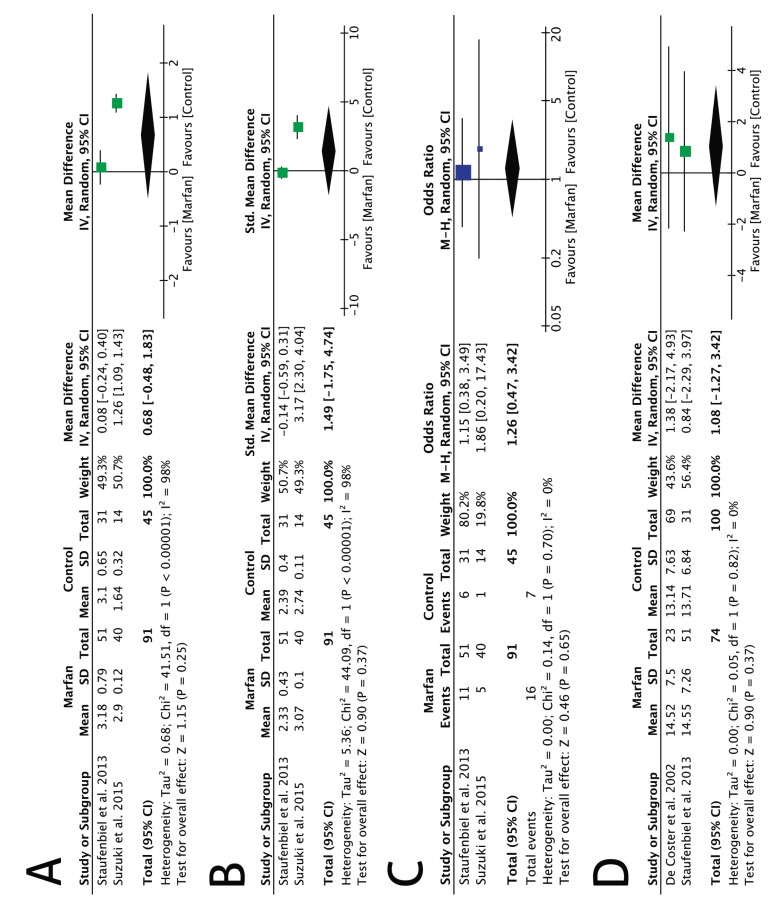
Forest plot for periodontal status score (mean difference) (A), probing depth (mean difference) (B), bleeding on probing (odds ratio) (C) and Decayed, Missing and Filled Teeth Index score (D) comparing patients with Marfan’s syndrome (exposed group) with healthy individuals (control group).

No statistically significant differences were found in periodontal status (MD: 0.68 points; 95% CI: -0.48 to 1.83; *P* = 0.25; I2: 98%) (Fig. 2A), PD (MD: -0.14 mm; 95% CI: -0.24 to 0.53; *P* = 0.46; I2: 93%) (Fig. 2B), BOP (OR: 1.26; 95% CI: 0.47 to 3.42; *P* = 0.65; I2: 0%) (Fig. 2C) or DMF-T score (MD: 1.08 points.; 95% CI: -1.27 to 3.42; *P* = 0.37; I2: 0%) (Fig. 2D).

## Discussion

To our knowledge, the present study, which used recommended methods for systematic reviews and meta-analyses, is the first that has quantitatively analyzed the relationship between MFS and oral health status.

Three analytical cross-sectional studies involving total or partially dentate participants evaluated if patients diagnosed with MFS had a higher risk of developing oral diseases compared to healthy individuals (8,9,21). Meta-analysis of these papers showed MFS groups were not associated with significantly worsened BOP rates, mean PD, periodontal status or DMT-T score (Figure 2). However, these results should be approached with caution since all four investigations had a potential risk of bias. In addition, their internal validity may have been compromised because longitudinal designs only allow us to establish a true cause and effect relationship. This issue could affect the reliability and quality of the studies. Furthermore, none of the papers included reported any sample size calculations. Given the small number of participants in all these studies, a type II error (failure to reject a false null hypothesis) may have occurred due to inadequate statistical power. Another possible limitation of this article is that due to the small number of papers available for review an evaluation of publication bias (i.e., funnel plot) could not be carried out (18).

Periodontal disease is initiated by microorganisms in the subgingival biofilm, and lifestyle risk factors, as well as systemic diseases, play a role in modifying the disease (22). Although some authors have suggested that connective tissue disorders, such as MFS, are related with a higher prevalence or more severe forms of periodontitis (10,21,23,24), others, as well as our results (Fig. 2A,B), failed to demonstrate such associations (8). A possible explanation for this difference may be attributed to the fact that periodontitis is a complex and multifactorial chronic disease (22). In this sense, individual risk factors such as smoking, diabetes, poor oral hygiene or nutrition may be far more critical than the disorder itself. Nevertheless, it seems reasonable that patients with MFS should be considered to be a population group more susceptible to inflammatory breakdown of periodontal tissues. Therefore, strict maintenance therapy for the prevention of periodontal diseases is of utmost importance since it can prevent the onset of these afflictions or attenuate their severity.

De Coster *et al.* (9) revealed a worse gingival index in MFS patients when compared to controls ( Table 4,  Table 4 continue). To the contrary, when a more objective method such as BOP was used to assess periodontal inflammation, no significant differences were found between the groups (Fig. 2C). One explanation for this may be that other variables could act as confounders. In this regard, crowded teeth are usually found in subjects with MFS (8,23). Undeniably, maintaining a proper oral hygiene can be challenging in these cases. Therefore, the higher degree of inflammation in patients with MFS may have been the result of the malocclusion rather than a true manifestation of the disorder (8).

Dental examination of patients with MFS can reveal the presence of local spots of hypoplastic enamel, root deformity, abnormal pulp shape and pulpal inclusions (9). However, no differences were found between groups in terms of DMF-T score (Fig. 2D) according to the results of our metanalysis.

It has been claimed that Marfan patients have a higher risk of developing bacterial endocarditis (25). Although the present study failed to find significant differences between groups, the prevention of bacteremia caused by advanced tooth decay, pulpal infection and/or periodontal diseases should be given high priority in dental treatment planning in MFS patients, especially in those who wear prosthetic heart valves or other devices for treating the syndrome’s cardiovascular complications of the syndrome. Therefore, an adequate dental monitoring as well as a strict maintenance therapy program should be implemented.

The goal of a systematic review is not only to qualify and synthesize the scientific evidence, but also to map out and categorize the existing literature on a particular topic (26). This is undertaken in order to characterize the quantity and quality of available information and based on this new knowledge to provide recommendations for future investigations as bias-free as possible (27). Accordingly, researchers are encouraged to examine the effect of MFS as an independent risk factor in the development, progression, and severity of oral diseases compared to completely healthy subjects in order to validate or refute our findings. Future research should therefore be based on longitudinal studies in order to detect developments or changes over a long period of time focusing on the characteristics of the target population at both the group and the individual level.

## Conclusions

According to the results of our meta-analysis, MFS is not associated with worsened oral health status. Nevertheless, since only three analytical cross-sectional studies were included, longitudinal designs are needed to establish a true cause and effect relationship. Moreover, due to the high number of MFS patients with prosthetic heart valves, an adequate dental monitoring as well as a strict maintenance therapy program should be implemented in order to prevent the onset or to attenuate the severity of oral diseases.
